# Arterial embolization in the treatment of multiple renal and hepatic hamartomas with spontaneous hemorrhage and 2-year follow-up: a case report

**DOI:** 10.1186/s13256-024-04368-8

**Published:** 2024-04-16

**Authors:** Jianhua Zhang, Tao Zhen, Hongmei Jian, Jinlan Yang, Ni Zhang

**Affiliations:** 1 Department of Oncology, The Second Affiliated Hospital of Chongqing Medical University, Chongqing, China; 2 Department of Interventional Radiology, Fengjie County People’s Hospital, Chongqing, China; 3 Department of Oncology, Fengjie County People’s Hospital, Chongqing, China

**Keywords:** Multiple hamartomas, Arterial embolization, Hemorrhage, Follow-up, Case report

## Abstract

**Background:**

Hamartoma is a common benign tumor that usually occurs in the kidney, liver, lung, and pancreas. Large renal hamartomas may spontaneously rupture and hemorrhage, which is potentially life-threatening.

**Case presentation:**

This report describes a 46-year-old Han Chinese female patient with multiple renal and hepatic hamartomas with rupture and hemorrhage of giant hamartoma in the left kidney. She underwent arterial embolization three times successively, and her condition was stable during the 2-year follow-up. This report includes a review of the relevant literature

**Conclusions:**

the findings in this report and previous literature suggest that arterial embolization can not only rapidly treat hamartoma hemorrhage in the acute phase but can also effectively control multiple lesions in the long term after repeated multisite arterial embolization.

**Supplementary Information:**

The online version contains supplementary material available at 10.1186/s13256-024-04368-8.

## Introduction

Hamartoma belongs to a family of neoplasms, named epithelioid angiomyolipoma/pure epithelioid (PEComa). Although hamartoma is the most common benign tumor, worrisome features may be observed, such as the increase of tumor volume and the rupture and bleeding of the tumor. Significant mortality has been associated with hamartoma due to its complications, and thus, medical therapy, angiographic arterial embolization, and surgical excision could also be necessary for the patient. Here, we report a patient with multiple renal and hepatic hamartomas with spontaneous hemorrhage. This case is deserving of being reported for the following reasons: (I) the patient was diagnosed with multiple renal and hepatic hamartomas without tuberous sclerosis, (II) the patient underwent emergency arterial embolization after rupture of a giant hamartoma in the left kidney and the bleeding stopped, and (III) effective control of hamartomas with only arterial embolization for approximately 2 years. We present the following article in accordance with the CARE reporting checklist (available at https://atm.amegroups.com/article/view/10.21037/atm-22-67/rc) (Additional file 1).

## Case presentation

**Fig. 1:**
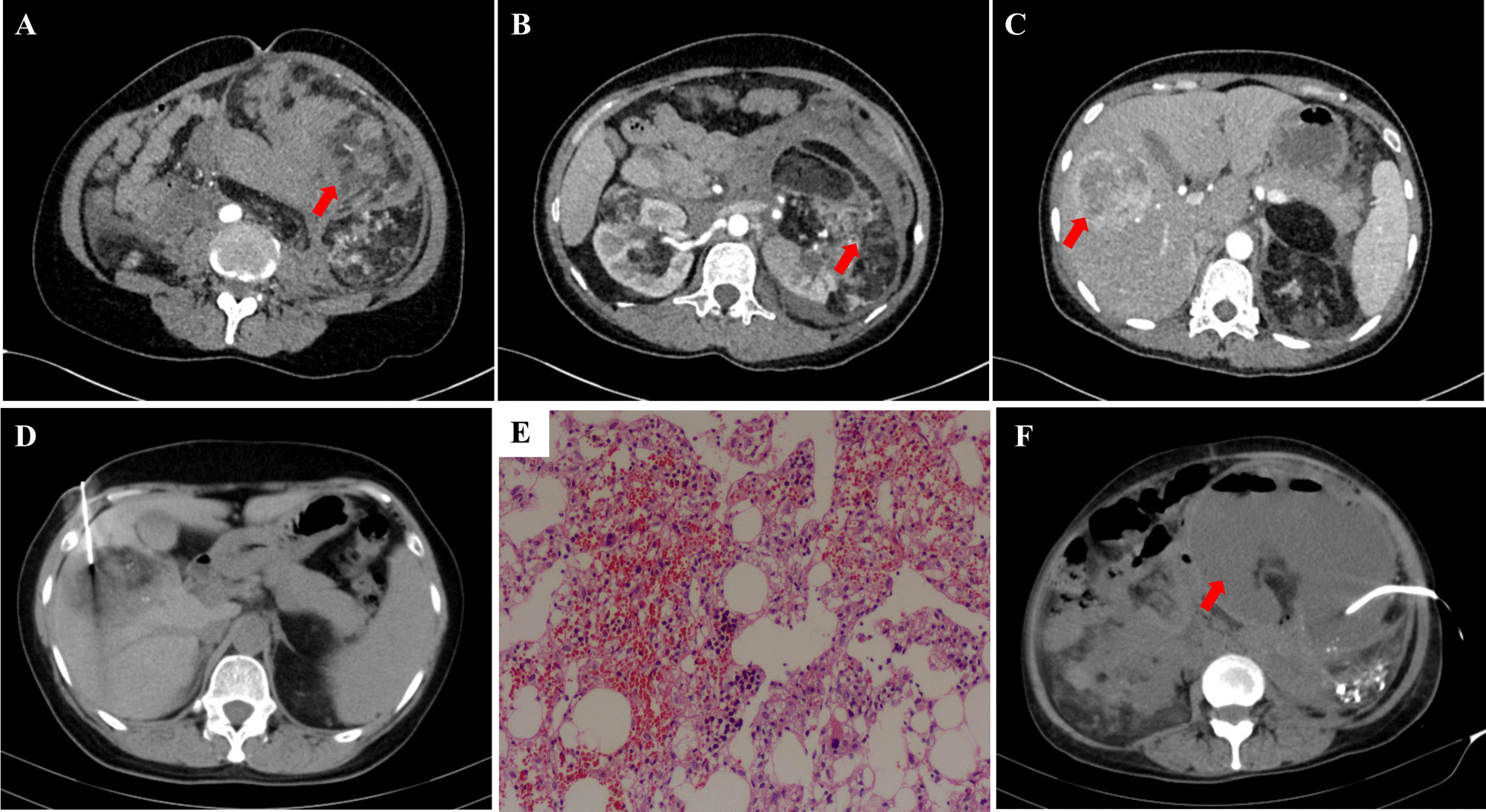
**A** CT image indicating a giant soft tissue shadow and a fat density shadow with blood accumulation in the left kidney (arrow). **B** Renal parenchyma enhancement during the arterial phase with multiple lesions in the bilateral kidneys (arrow). **C** CT imaging of the liver lesion in the arterial phase (arrow). **D** CT image of percutaneous biopsy of the liver lesion. **E** histopathological image of liver hamartoma demonstrating that the lesion was composed of well-differentiated adipose tissue, smooth muscle and thick-walled vessels (hematoxylin and eosin staining, × 200). **F** CT image of the drainage for left renal hamartoma abscess

A 46-year-old Han Chinese female patient was admitted to Fengjie County People’s Hospital (Chongqing, China) with the complaint of intermittent abdominal pain for 2 days in November 2020. No special personal medical and psychosocial history, no family history of genetic disorders, and 2 daughters born in natural birth. She was weak, anorexic and anemic with episodes of nausea and vomiting. Physical examination revealed tenderness over the left kidney and left lower abdominal. A giant mass ~ 10 × 10 cm was palpated in the left abdomen. Contrast-enhanced computed tomography (CT) scan revealed destruction of the bilateral renal structures, possible hamartoma, hemorrhage of the left renal lesion (Fig. 1A, B), and fluids in the pelvic cavities. In addition, multiple abnormally enhanced foci were observed in the liver, which were suspected to be hamartoma (Fig. 1C). Subsequently, the patient underwent a biopsy of the liver lesion under CT localization (Fig. 1D), and the pathology indicated angiomyolipoma (Fig. 1E).

**Fig. 2:**
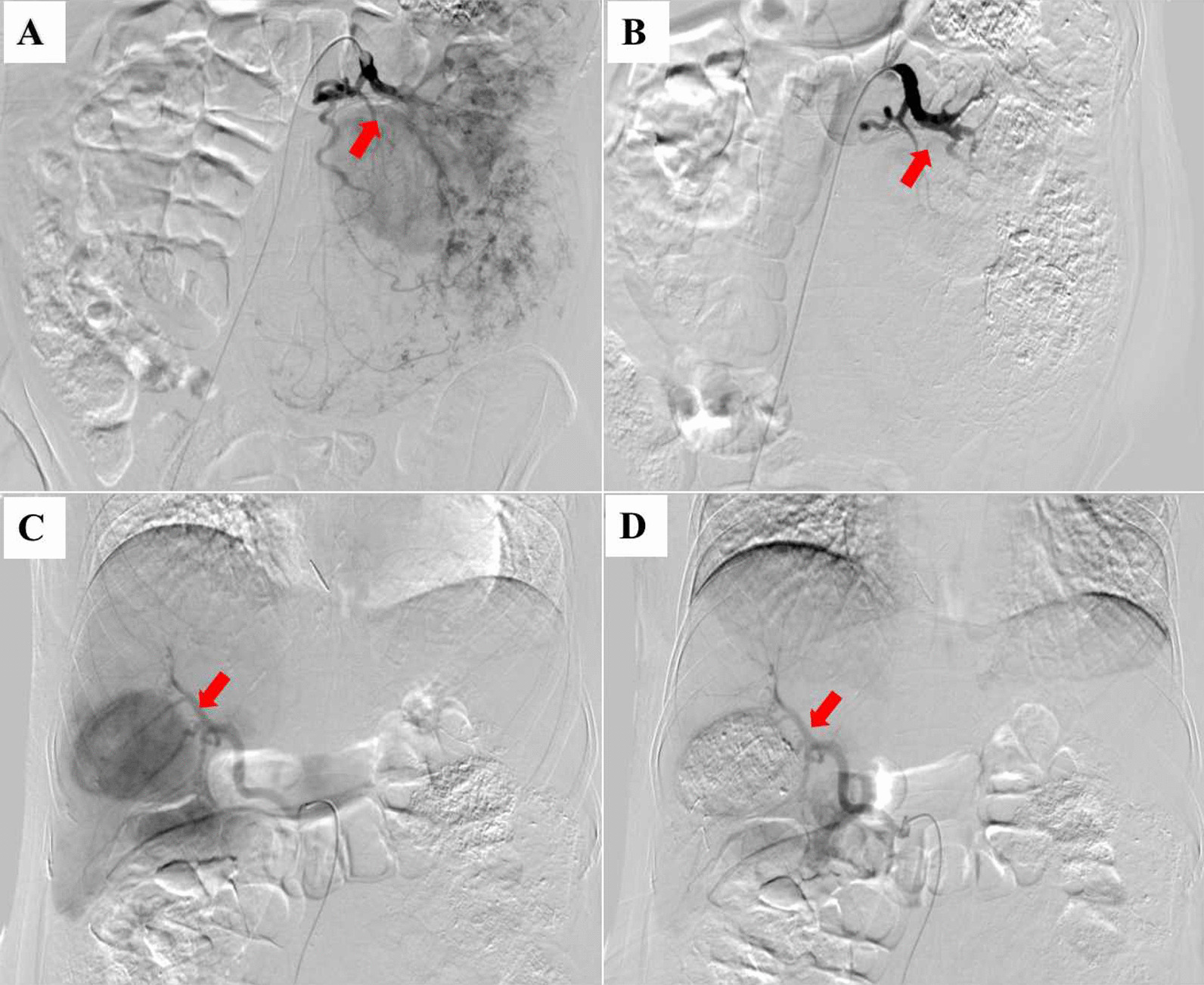
DSA images of left renal hamartoma before (**A**) and after (**B**) arterial angiographic embolization. DSA images of hepatic hamartoma before (**C**) and after (**D**) arterial angiographic embolization. Arrows indicate the hamartoma blood supply arteries blocked by interventional embolization

Emergency transcatheter renal arterial embolization and hepatic arterial embolization were performed under local anesthesia. The interventional procedure is shown below. The catheter sheath (RS*A50K10SQ, TERUMO) was inserted into the right femoral artery percutaneously, and the catheter and microcatheter (MC-PE27131, TERUMO) were implanted into the tumor-supplying branch of the left renal artery under a guide wire (RF*GA35153M, TERUMO). Digital subtraction angiography (DSA) showed that the tumor area of the left kidney had developed, and a small amount of contrast agent had spilled (Fig. 2A). Under X-ray fluoroscopy, the suspension of lipiodol and bleomycin (1:1, 14 ml) was slowly injected into the artery feeding left renal tumor. Then, a polyvinyl alcohol foam embolization of microparticles (350–560 μm cook, 1.5 vial) was slowly injected into the artery. DSA showed that the left renal tumor was not redeveloped and that lipiodol deposition in the tumor was obvious (Fig. 2B). Then, the catheter and microcatheter were implanted into the common hepatic artery, and DSA showed the development of a tumor in segment V of the liver (Fig. 2C). The embolization of the liver lesion was the same as the previous steps. DSA showed that the intrahepatic tumor was not redeveloped and that lipiodol was well deposited (Fig. 2D). The patient recovered well after the operation, and the abdominal pain was relieved.

The patient was readmitted to the hospital due to “fever and chills” 3 months later. Contrast-enhanced CT of the abdomen showed a left renal hamartoma abscess and perirenal infection. The patient underwent transabdominal abscess puncture and catheter drainage, which resulted in the discharge of brown, foul-smelling pus (Fig. 1F). Bacterial culture of pus suggested Escherichia coli. The patient then received abscess flushing and anti-infective treatment with piperacillin/tazobactam.

**Fig. 3:**
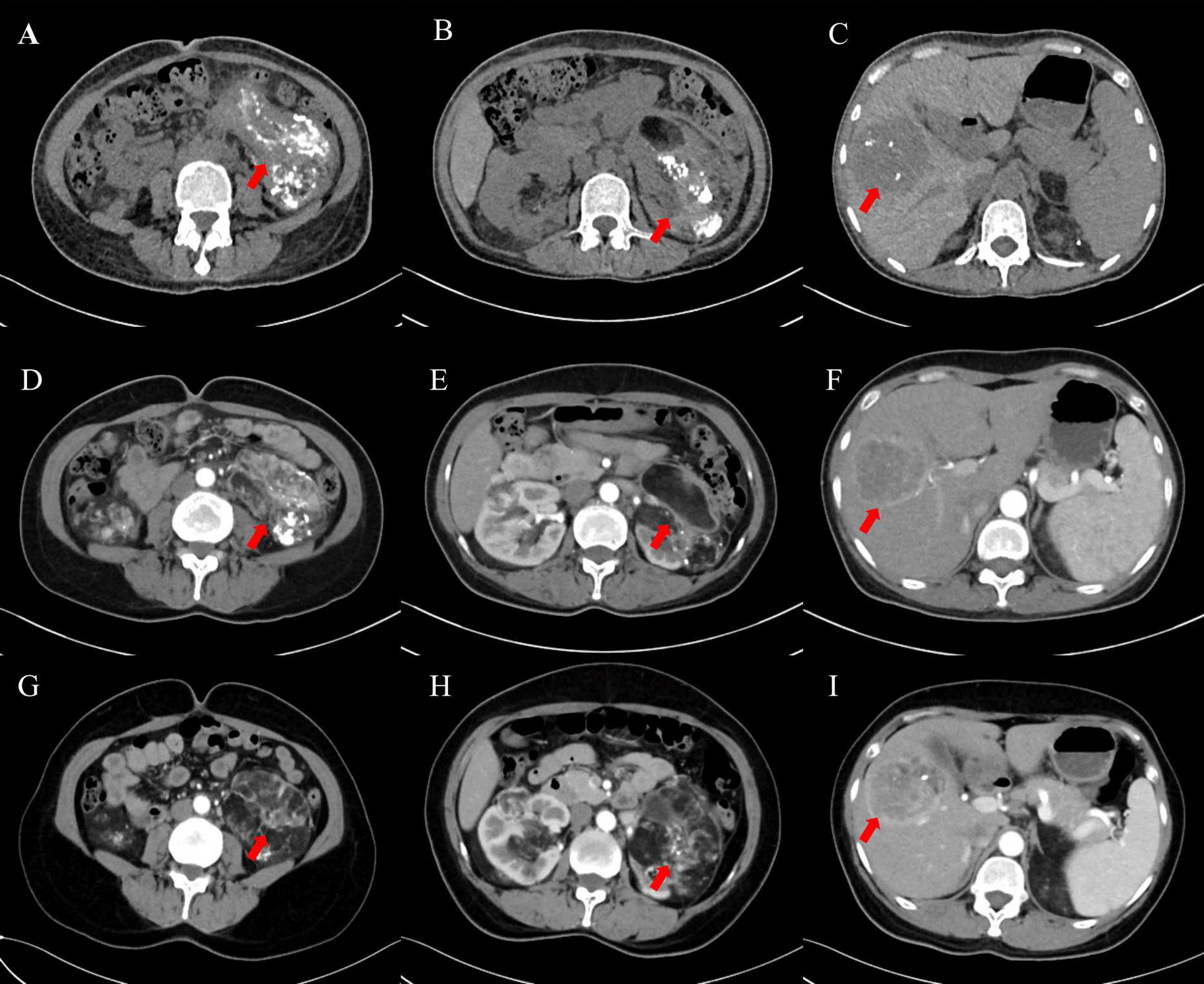
CT images of the patient during follow-up. **A**–**C** CT images obtained at 4-month follow-up. **D**–**F** CT images obtained at 6-month follow-up. **G**–**I** CT images obtained at 18-month follow-up. The arrows in **A**, **B**, **D**, **E** represent kidney lesion locations. The arrows in **C**, **F** represents the location of the liver lesion

**Fig. 4:**
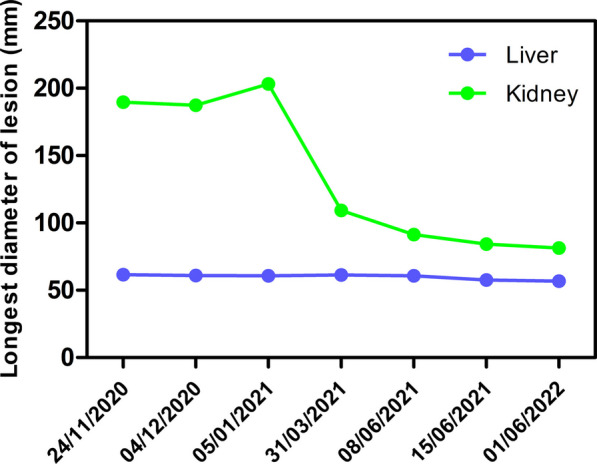
Graph of changes in the maximum diameter of kidney and liver hamartomas during treatment. The graph shows that the diameter of kidney hamartomas gradually decreases, while the diameter of liver hamartomas remains stable

Two more transcatheter renal arterial embolizations and hepatic arterial embolizations were performed on the patient in March 2021 and June 2021. The interventional surgery procedure was the same as before. Efficacy was evaluated as partial remission (PR) for the kidney foci and stable disease (SD) for the liver foci (March 2021, June 2021 and June 2022) compared to CT on November 2020. The images of the patient’s successive evaluations during the treatment are shown in Fig. 3A–I. The maximum tumor diameter for both the kidney foci and liver foci during the patient’s treatment appeared to decrease significantly during the treatment, as shown in Fig. 4. Written informed consent was obtained from this patient for publication of this case report and accompanying images.

## Discussion

Hamartoma, also known as angiomyolipoma, is a benign tumor composed of mature adipose tissue, smooth muscle, and thick-walled blood vessels [1]. Hamartoma may occur in any organ, such as the gastrointestinal tract and respiratory system, but it most frequently occurs in the kidneys [2, 3]. Renal angiomyolipoma (RAML) has been reported sporadically in patients with tuberous sclerosis. Tuberous sclerosis a hereditary syndrome that is caused by alterations in the TSC1 (9q34) or TSC2 (16p13.3) genes, and it predisposes patients to several manifestations, including mental retardation, seizures, cardiac rhabdomyomas, and hamartomas [4]. Sporadic RAML is more common in adults, with an incidence of approximately 0.13% and a male-to-female ratio of approximately 1:7 [5]. To the best of our knowledge, multicentric hepatic and renal hamartomas have rarely been reported in sporadic hamartoma populations. This report provides a case of massive multiple hamartomas of the liver and kidney in a female patient without tuberous sclerosis.

Hamartoma may be diagnosed by ultrasonography, CT, and MRI. Wang *et al.* described the radiological features of hamartomas [6, 7]. Ultrasound findings usually include a hyperechoic mass with or without cystic areas or calcifications. Color Doppler usually shows a hyper vascular mass. However, it is difficult to differentiate giant renal hamartoma from other retroperitoneal fatty lesions by ultrasound alone. According to previous literature [7, 8], most low-fat hamartomas exhibit high density or slightly high density on CT scans, and a few demonstrate slightly low density. The hamartoma is slightly hypodense before contrast administration. After contrast injection, prolonged enhancement is achieved in a mass. In this report, CT image of the renal lesion was consistent with typical hamartoma, such as popcorn-like calcified shadows and fatty shadows. However, the liver lesion was atypical, so we performed a biopsy of the liver lesion and found that it was still hamartoma. MRI findings include an isointense mass in T1-weighted images and a hyperintense mass in T2-weighted images [9]. Although MR is more sensitive than CT for the evaluation of liver foci, CT was chosen in this case for financial consideration. Finally, diagnosis is confirmed by histopathological examination and is verified by immunohistopathology. The melanogenesis markers (HMB45 and Melan-A) and cathepsin K are extremely helpful in the diagnosis [10, 11].

Generally, hamartomas are asymptomatic. Only large lesions can manifest clinical symptoms, such as palpable masses and spontaneous intra-abdominal rupture. It is well known that large hamartomas are prone to life-threatening hemorrhage into the retroperitoneum (Wunderlich’s syndrome). This clinical symptomatology seems to occur more often in women. Pregnancy may promote tumor growth and increase the risk of tumor rupture, which previous studies hypothesized was due to the ubiquitous expression of estrogen and progesterone receptors in RAML. With the elevation of estrogen and progesterone levels in pregnant women, the tumor tends to grow quickly under stimulation of tumor vascular proliferation. This increases the risk of spontaneous rupture and bleeding of the tumor or surrounding structures [12, 13]. Besides, renal failure is another important complication when multiple RAML occur, a condition more frequent in patients with tuberous sclerosis.

Although typical hamartomas are benign mesenchymal neoplasms, early detection and diagnosis of hamartomas may decrease the occurrence of serious complications. For unruptured hamartomas > 4 cm, minimally invasive procedures, including arterial embolization, radiofrequency ablation, cryoablation and partial or total nephrectomy, and other novel therapies, such as mTOR inhibitors, are recommended [14–16]. The activation of the mTOR pathway due to genetic alterations of the tuberous sclerosis complex in the TSC1 or TSC2 genes in hamartomas has also been reported as well as the subsequent therapeutic implications [17]. While mTOR inhibitors are clinically important to reduce hamartoma size, their volume tends to increase after the treatment is stopped. However, nephron-sparing surgery (NSS) is widely regarded as the best surgical procedure in clinical practice because of its feasibility and effectiveness. For both open surgery and laparoscopic surgery, the main disadvantages are a certain rate of nephrectomy, large blood loss, large trauma, and slow recovery [18, 19]. Although advanced surgical techniques, such as robotic partial nephrectomy, have dramatically reduced surgical complications, the accessibility is relatively low in underdeveloped areas. Furthermore, renal arterial embolization has recently been used as first-line treatment for acute RAML rupture and is increasingly used to prevent bleeding-prone RAML [20]. For lesions residual after embolization, surgical removal is usually recommended in the clinical practice. Considering the wide range of surgery, the patient opted for follow-up embolization and observation. However, selective renal arterial embolization still has certain complications, such as fever, nausea and vomiting, gastrointestinal symptoms, and hematuria [21]. In this report, the patient presented with a giant hamartoma with necrosis and infection after arterial embolization. Finally, the indications for arterial embolization include hemostatic treatment of acute phase hemorrhage and prophylactic hemostasis, such as ruptured hemorrhage of substantial organs, hemoptysis, and acute obstetric hemorrhage. The advantages of interventional hemostasis compared to conventional procedures are minimally invasive, reproducible, precise, and efficacious. At present, the patient's condition is gradually improving, and postoperative follow-up is important and necessary.

## Conclusion

To improve the survival rate of patients with hamartoma, early imaging diagnosis and follow-up are important. When the diagnosis is confirmed, interventions including renal arterial embolization should be considered. At present, for RAML with spontaneous rupture and hemorrhage, arterial embolization appears to be an effective treatment to save the life of the patient.

### Supplementary Information

**Additional file 1.** CARE Checklist of information to include when writing a case report.

## Data Availability

Not applicable.
